# Inferring activity changes of transcription factors by binding association with sorted expression profiles

**DOI:** 10.1186/1471-2105-8-452

**Published:** 2007-11-16

**Authors:** Chao Cheng, Xiting Yan, Fengzhu Sun, Lei M Li

**Affiliations:** 1 Molecular and Computational Biology Program, Department of Biological Sciences, University of Southern California, Los Angeles, CA 90089-2910, USA; 2 Department of Mathematics, University of Southern California, Los Angeles, CA, 90089-2532, USA

## Abstract

**Background:**

The identification of transcription factors (TFs) associated with a biological process is fundamental to understanding its regulatory mechanisms. From microarray data, however, the activity changes of TFs often cannot be directly observed due to their relatively low expression levels, post-transcriptional modifications, and other complications. Several approaches have been proposed to infer TF activity changes from microarray data. In some models, a linear relationship between gene expression and TF-gene binding strength is assumed. In some other models, the target genes of a TF are first determined by a significance cutoff to binding affinity scores, and then expression differentiation is checked between the target and other genes.

**Results:**

We propose a novel method, referred to as BASE (binding association with sorted expression), to infer TF activity changes from microarray expression profiles with the help of binding affinity data. It searches the maximum association between bind affinity profile of a TF and expression change profile along the direction of sorted differentiation. The method does not make hard target gene selection, rather, the significances of TF activity changes are evaluated by permutation tests of binding association at the end. To show the effectiveness of this method, we apply it to three typical examples using different kinds of binding affinity data, namely, ChIP-chip data, motif discovery data, and positional weighted matrix scanning data, respectively. The implications obtained from all three examples are consistent with established biological results. Moreover, the inferences suggest new and biological meaningful hypotheses for further investigation.

**Conclusion:**

The proposed method makes transcription inference from profiles of expression and binding affinity. The same machinery can be used to deal with various kinds of binding affinity data. The method does not require a linear assumption, and has the desirable property of scale-invariance with respect to TF-specific binding affinity. This method is easy to implement and can be routinely applied for transcriptional inferences in microarray studies.

## Background

Transcription factors (TF) play a central role in many critical biological processes, such as transcriptional regulation, cell proliferation, development, and apoptosis. During signal transduction, the extra- or intra-cellular signals are conveyed eventually to certain transcription factors, leading to their activation or repression and consequently changing the expression of their target genes. Thus, the identification of transcription factors associated with a biological process is fundamental to understanding its regulatory mechanism.

DNA microarray technology has been widely applied to functional genomic studies, in which mRNA expression levels for thousands of genes are measured simultaneously. In a typical experimental design, gene expressions are measured for a collection of samples from two classes, e.g. tumor versus normal tissues. After appropriate processing of microarray data, we can obtain the mRNA expression change of every gene between the two classes. For transcription factors, however, it is often difficult to infer their activity changes based only on their own mRNA expression levels for the following reasons: (1) the mRNA expression levels of many TFs are often relatively low compared to other genes; (2) the activities of TFs are prevalently regulated by post-transcriptional modifications, e.g. protein phosphorylation, which cannot be captured by gene expression microarrays; (3) other complications in regulation may also exist.

To overcome these challenges, several approaches have been proposed in the literature to infer TF activities through expression changes of their regulated target genes. These approaches can roughly be divided into two classes according to the type of binding affinity data used for inference. The first class, including REDUCE [1] and MOTIF REGRESSOR [2], identify regulatory motifs (putative TF binding sites) associated with gene expression changes. The second class make use of the ChIP-chip data, which provide direct experimental binding information of TFs with genomic sequences [3]. This class includes the network component analysis (NCA) introduced by Liao *et al. *[4], the pseudo-inverse projection method described by Alter *et al. *[5], the MA-Networker algorithm proposed by Gao *et al. *[6], and the partial least squares (PLS) regression method suggested by Boulesteix *et al. *[7]. Common to these approaches, a linear relationship between gene expression changes and TF-gene binding affinities is assumed. The two motif-based methods, REDUCE and MOTIF REGRESSOR, also assume a linear relationship between expression changes and motif occurrences (REDUCE) or motif matching-scores (MOTIF REGRESSOR) in the upstream regions of genes. Unfortunately, the linear relationship may not be valid considering the high complexity of gene transcription regulation. Tsai *et al. *proposed a statistical method to identify cell cycle associated TFs in yeast, which used the Kolmogorov-Smirnov (KS) test to examine whether expressions of the target and non-target gene sets of a TF are significantly different [8]. This method does not assume a linear relationship between gene expression changes and TF-gene binding affinities, whereas a threshold value, which is more or less arbitrarily selected, must be specified to determine the target and non-target gene sets for a TF.

**Figure 1:**
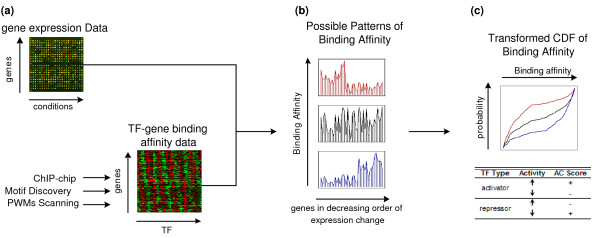
**The schematic representation of our method**. **(a) **The inputs are the gene expression data and the binding affinity data such as ChIP-chip data, motif discovery data or PWMs scanning data. **(b) **The possible patterns of binding affinities of ranked genes in a decreasing order of their expression changes. The top red and bottom blue plot represent cases where the up-regulated (red) or down-regulated (blue) genes tend to have high binding affinity by a TF. The middle plot (black) shows when there is no significant correlation between the gene expression data and the binding affinity data. **(c) **The presentation of the binding affinity patterns using cumulative distribution functions. The binding affinity patterns shown in (b) is regarded as probability density functions. The lower table shows the relationship between TF activity change and the expression change of the target genes.

In this article, we propose a new method, referred to as BASE (binding association with sorted expression), to infer TF activity changes by integrating microarray expression data with binding affinity data such as ChIP-chip data or motif data. The basic idea of the method is illustrated in Figure 1. In general, activity change of a TF can be reflected by expression changes of its target genes in the microarray data. Given a sorted expression change profile, we would observe different binding affinity patterns for TFs with enhanced, reduced, or unchanged activities as shown in Figure 1(b) and 1(c). It should be noted that the association between TF-gene binding affinities and target gene expression changes may exist only in a local region (e.g., most up- or down-regulated region) rather than across all genes. This local association is difficult to be detected by standard linear methods. In contrast, BASE is designed to detect the local association between TF-gene binding affinities and gene expression changes to increase the power of transcriptional inference. We illustrate the method by three case studies using different types of binding affinity scores: ChIP-chip data, motif discovery data, and positional weighted matrix (PWM) scanning data. In these data sets, our methods achieve results that are biologically meaningful and consistent with previous studies.

## Results and Discussion

We demonstrate the ability of our method to provide biological meaningful insights using three examples for which considerable background information is available. For the first example, we combine ChIP-chip data with microarray data from transcription factor perturbation experiments (TFPE) that measure gene expression changes in TF-deleted or TF-overexpressed yeast strains with respect to the wild-type. For the second example, we integrate gene expression data with motif discovery data to identify transcription factors that may account for the life span extension in three long-lived yeast mutants. For the third example, gene expression data is integrated with the positional weight matrices (PWM) information to detect transcription factors that are activated or repressed in three subtypes of human lung tumors.

### TF activity changes in TFPE microarray data

We collect 76 microarray gene expression profiles from previous TFPE in yeast, including 27 deletions and 49 over-expressions of transcription factor [9-12]. We combine these 76 microarray gene expression profiles with ChIP-chip data to identify the activated or repressed TFs in these TFPEs. The ChIP-chip data is from the systematic experiments performed by Harbison *et al. *[3], where genomic occupancies of 203 yeast TFs in YPD medium were measured. For some of these TFs, genomic occupancies in several other environmental conditions were also determined, such as heat shock and rapamycin treatment. We calculate the activity change scores (AC scores) as well as their significances (refer to the Methods section) for each combination of the 76 gene expression change profiles and the 350 ChIP-chip profiles (203 in YPD condition and 147 in the other conditions).

Since ChIP-chip experiments are carried out for all the 203 TFs only in the YPD medium, our inference first focuses on the ChIP-chip data under this condition. Our results show that in 20 out of 27 TF deletion and 30 out of 49 TF over-expression TFPEs, the known perturbed TFs are found to be substantially activated or repressed at the 0.01 significance level (q-value < 0.01, see Additional file 1 and file 2). It should be noted that deletion or over-expression of a TF may not always cause expression changes of its target genes [13]. First, different TFs often form a certain complex to regulate transcription and thereby over-expression or removal of a single component of the complex may not lead to apparent expression changes of its target genes. Second, if the activity of a TF in the wild-type is inherently high/low, over-expression/deletion of the TF may not substantially change its target gene expression. Finally, many confounding factors such as function redundancy and post-translational modifications may exist.

Regardless of the complications, when a TF is deleted, by and large we would expect down-regulation of the target genes if it is a transcriptional activator, or up-regulation of the target genes if it is a transcriptional repressor. Conversely, when a TF is over-expressed, we would expect to observe the opposite expression changes of its target genes. Among these perturbed TFs in the TFPEs, the majority are transcriptional activators and only 5 (Hir2, Mbp1, Bye1, Gzf3 and Rox1) function as transcriptional repressors according to previous studies [14-18]. The activity inferences of these 5 repressors are consistent with what are expected: the AC scores for Hir2 in *hir2Δ *and Mbp1 in *mbp1Δ *are 18.2 (q-value = 0) and 15.9 (q-value = 0), respectively, suggesting the up-regulation of their target genes; whereas in their over-expressed strains the AC scores for Bye1, Gzf3, Mbp1 and Rox1 are -9.8 (q-value = 0), -5.9 (q-value = 0.0027), -12.1 (q-value = 0) and -6.8 (q-value = 0.0001), respectively, suggesting the down-regulation of their target genes. For the remaining perturbed TFs which are known as transcription activators, the inferred activity changes of them are also consistent with our expectations (see Additional File 1 and File 2), but with a few exceptions. These exceptions may imply more delicate mechanism regarding TF activity regulation. For example, rather than a positive value, the AC score of Met4 (a transcriptional activator) is -13.2 (q-value = 0) in its over-expressed strain. This inconsistency may reflect the difference of Met4 at expression and activity levels, since it has been reported that Met4 controls its own degradation through a negative feed back loop [19,20]. Alternatively, these exceptions may also be caused by incomplete TF functional annotation or by the difference in experimental conditions between the micorarray and ChIP-chip experiments. It is possible that for some TFs, the TF-gene binding affinities in the ChIP-chip data do not match the true regulatory relationship under the microarray experiment condition.

In addition, our results indicate that deletion or over-expression of a TF can lead to activity change of some other TFs, suggesting either regulatory relationships between these TFs or a significant overlapping of their target genes. For example, there are 7 and 28 other TFs that are significantly changed in activity in *mbp1Δ *and *gcn4Δ*, respectively. Further investigation of these regulatory relationships may be helpful to understand the TF-TF interaction during transcriptional regulation.

We next examine the inferred TF activity changes based on the ChIP-chip data under all the available conditions: YPD (rich nutrient medium), H2O2Hi (highly hyperoxic, 4 mM H2O2), H2O2Lo (moderately hyperoxic, 0.4 mM H2O2), SM (amino acid starvation, 0.2 mg/ml sulfometuron methyl), Acid (acidic medium, 0.05 M succinic acid), RAPA (nutrient deprivation, 100 nM rapamycin) and BUT14 (filamentation inducing, 1% butanol). It turns out that for some TFs, when the ChIP-chip data under different conditions are used, the inferred activity changes in a given microarray experiment can vary substantially. Let us use Yap7 in *yap7Δ *as the example: based on ChIP-chip data from YPD medium, the inferred AC score is -9.8 (q-value = 0.0002); while based on ChIP-chip data from H2O2Hi treatment, the inferred AC score is 10.4 (q-value = 0). This conflict results from the dynamic nature of association between TFs and genes.

According to the ChIP-chip experiments, some TFs including Yap7 may associate with a different set of genes under different cell status, medium, or other conditions [3]. Further computation shows no significant correlation between the binding profiles of Yap7 under the YPD and H2O2Hi conditions: the Spearman correlation coefficient is 0.002. Therefore, when combining microarray data with ChIP-chip data to infer TF activities, we should be cautious of the conditions under which the microarray and ChIP-chip experiments are performed. If the two experiments are performed under the same or similar conditions, the activity change inferences are reliable. Otherwise, the inferences are reliable only for those TFs that bind to invariant sets of genes under different conditions.

**Figure 2:**
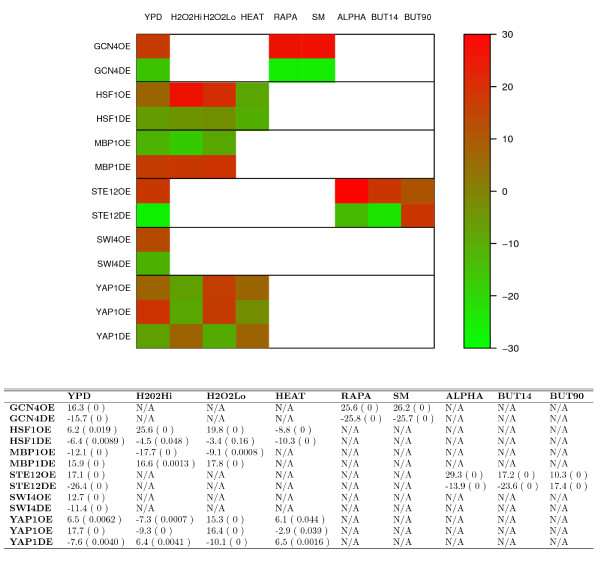
**Comparison of inferred AC scores for 6 TFs in their corresponding over-expression and deletion TFPEs**. The upper image shows the AC scores of Gcn4, Hsf1, Mbp1, Ste12, Swi4 and Yap1 under different combinations of the expression profiles and ChIP-chip data. The lower table shows the AC scores as well as the significance levels(in bracket). Each row represents the expression profile when the corresponding TF is over-expressed (OE) or deleted (DE). The columns correspond to all conditions under which the ChIP-chip data for the corresponding TF is measured. N/A means not available, which is due to the unavailability of the ChIP-chip data under the conditions for the TF. Note there are two rows for YAP1OE, each corresponding to an independent expression profile from Yap1 over-expression experiment.

**Figure 3:**
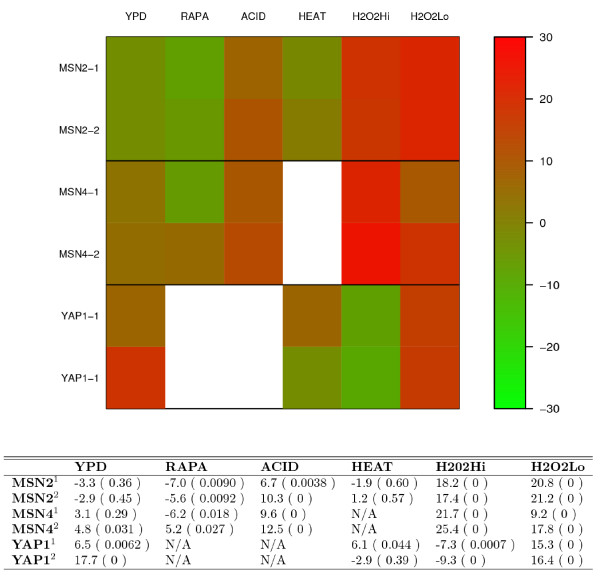
**Consistency of inferred AC scores for TFs in the corresponding over-expression TFPEs**. The upper image shows the AC scores for Msn2, Msn4, and Yap1 inferred from two independent over-expression TFPE data in combination with ChIP-chip data from different conditions. The lower table shows the AC scores as well as the significance levels (in bracket). Rows are different TFPEs for the corresponding TF and columns are different conditions under which the ChIP-chip data for the corresponding TF is measured. The superscript in the first column distinguishes the two independent gene expression profiles. N/A means not available, which is due to the unavailability of the ChIP-chip data for the TF under the condition.

Both deletion and over-expression TFPE data are available for 6 TFs: Gcn4, Hsf1, Mbp1, Ste12, Swi4 and Yap1, so we examine the consistency of activity inferences for these TFs in the deletion and over-expression TFPEs. As shown in Figure 2, in all except two cases, our method achieves consistent results for TF activity inference. For example, Gcn4, the transcriptional activator of amino acid biosynthetic genes, is inferred to be activated in Gcn4 over-expressed yeast strain (the AC scores are 16.3, 25.6, and 26.2 under YPD, RAPA, and SM condition, respectively) and repressed in *gcn4Δ *strain (the AC scores are -15.7, -25.8, and -25.7 under YPD, RAPA, and SM condition, respectively). Moreover, over-expressed TFPEs for Msn2, Msn4, and Yap1 have been performed independently by two research groups [11,12]. We examine consistency of the activity inferences from both data sets. As shown in Figure 3, our method achieves similar results for the two independent microarray expression data sets. It should be noted that expression profiles from the two Yap1 over-expression microarray experiments are not significantly similar with each other (the Spearman correlation coefficient is 0.02), perhaps due to high noise introduced during microarray experiments. Nevertheless, the transcriptional inferences for Yap1 from both data sets are still in good consistency, suggesting the robustness of our method to noise in gene expression data.

We also apply the method proposed by Tsai *et al. *[8] to these 76 TFPE microarray profiles. Based on the ChIP-chip data, a target gene set (p-value < 0.01) and a non-target gene set (p-value > 0.8) are defined for each TF, and expressions of genes in these sets are compared using the Kolmogorov-Smirnov test. As shown in Additional file 3 and file 4, this method detects activity changes of 14 TFs from their TFPEs (5 out 27 in the deletion strains and 9 out of 49 in the over-expression strains), all of which are identified by our method. This suggests that our method is more sensitive, since the BASE method examines the maximum local association between the expression change profile and the TF-gene binding affinity profile. According to our observations, mostly the association between these profiles only exists at the two end regions in the sorted expression change profile (e.g. the most up- or down-regulated region). It is hard to detect these local associations if we examine correlations across all genes, as the linear regression based methods do. In fact, for each of the 76 TFPEs, we calculate the Pearson correlation coefficient between its expression change profile and its binding affinity profile of the corresponding TF and it turns out that only 8 TFPEs have a correlation greater than 0.10.

### TF activity changes in long-lived yeast mutants

Recent studies suggest that three nutrient responsive kinases: Sch9, PKA, and TOR, may play important roles in yeast ageing. For example, inactivation of Sch9 kinase increases the replicative life span (the total number of daughter cells generated by a mother cell) by 30–40% [21] and extends the chronological life span (the maximum survival time of a non-dividing cell population in liquid medium) by nearly three fold [22]. To understand the mechanism of ageing, we generate three long-lived yeast mutants: *sch9Δ*, *ras2Δ *and *tor1Δ*, in which Sch9, PKA, and TOR kinase are inactivated, respectively. Although the mechanisms of longevity in these mutants have not been fully understood, two stress response transcription factors, Msn2/4 and Gis1, are likely to be involved since deletion of Msn2/4 in *ras2Δ *and deletion of Rim15, a kinase that activates Gis1 in *sch9Δ *reverse the survival extension [22]. We measure the gene expressions of the three mutants and a wild-type yeast strain using microarrays and obtain three expression change profiles: *sch9Δ/wt*, *ras2Δ/wt *and *tor1Δ/wt*.

In what follows, we apply the BASE method to identify the TFs associated with longevity in these mutants by integrating microarray data with motif discovery data. First, 537 putative regulatory motifs are identified from the promoter regions of all yeast genes using AlignACE, a de novo motif discovery method [23]. We then scan the promoter region of each gene to examine their occurrences. For each of the 537 putative motifs, we end up with a matching-score vector, which measures the transcriptional potentials of the binding motif for genes. Finally, the matching-score vectors are combined with the expression change profiles (*sch9Δ/wt*, *ras2Δ/wt *and *tor1Δ/wt*) to identify the regulatory motifs associated with gene expression modification in the mutants.

The results of our computational inference indicate that the activities of 53, 57 and 74 motifs are significantly changed in *sch9Δ/wt*, *ras2Δ/wt*, and *tor1Δ/wt*, respectively (see Additional file 5). Among these 537 motifs, 42 can be associated with transcription factors according to literature and databases [24]. Table 1 shows 16 motifs of these 42 motifs, which have significant activity changes in at least one of the three long-lived mutants. As shown in the table, both Msn2/4 and Gis1 are found to be significantly activated in all three long-lived mutants. Although the experimental justifications are only available for Msn2/4 in *ras2Δ *and for Kim15 (Gis1 activator) in *sch9Δ *[22], our results suggest that in all three mutants, Msn2/4 and Gis1 may play a critical regulatory role in life span extension. Consistently, some studies have shown the negative regulation of Msn2/4 activity by PKA and TOR kinase as well as the negative regulation of Gis1 activity by PKA kinase [25-28]. In addition to Msn2/4 and Gis1, we identify other TFs with significant activity changes, such as Fhl1, Sum1/Ndt1 and Pho4, which may also be critical for life span extension and further investigations may shed new light on the mechanism of longevity in these long-lived yeast mutants.

**Table 1:** Motifs significantly associated with gene expression changes in long lived mutants, sch9Δ, ras2Δ, and tor1Δ, with respect to the wild-type yeast.

		*sch9Δ*/wt			*ras2Δ*/wt			*tor1Δ*/wt		
TF	Consensus	AC	p-value	q-value	AC	p-value	q-value	AC	p-value	q-value
Fhl1	RTGT-YGGRTG	17.0	0	0	12.4	0	0	16.0	0	0
Msn2/4	AGGGG	15.0	0	0	15.4	0	0	12.5	0	0
Gis1	AWAGGGAT	12.0	0	0	12.5	0	0	10.6	0	0
Sum1/Ndt80	GACACAAAA	8.7	0	0	9.3	0	0	9.2	0	0
Pho4	GY-TSKCACGTG-G	7.3	0.0006	0.0013	9.5	0	0	8.7	0.0006	0.0025
Pdr3	S-TCCGYGGAA	6.0	0.011	0.012	9.1	0.0006	0.0009	4.4	0.067	0.077
Cad1	ATTAGTAAGC	5.9	0.014	0.015	8.5	0.0006	0.0009	4.6	0.033	0.048
Mig1	CGCATMCCCCAC	5.2	0.037	0.029	7.8	0	0	5.6	0.053	0.066
Met31	MWGTGTGGCR	4.9	0.041	0.031	9.2	0	0	5.4	0.047	0.061
Hsf1	GAW-TTCTAGAA	4.3	0.16	0.083	9.1	0	0	4.6	0.087	0.091
Zap1	ACCYT-AGGTT	3.8	0.43	0.16	12.1	0	0	5.2	0.045	0.059
Met4	AMAA-TGTGG	3.4	0.45	0.16	9.8	0	0	5.8	0.054	0.067
Cbf1	RRTCACGTG	3.3	0.45	0.16	9.2	0	0	5.3	0.038	0.054
Swi4	CRCGAAAA	3.2	0.39	0.15	3.5	0.27	0.080	11.8	0	0
Abf1	CGT|-ARTGAT	-5.2	0.033	0.027	-9.3	0	0	-9.2	0	0
Xbp1	GCCTCGARGMGR	-7.1	0	0	4.4	0.24	0.075	-3.3	0.21	0.17

The 16 motifs that can be associated with a known TF and are significant in at least one of the three long-lived mutants are shown.

In a previous study, we identified motifs associated with life span extension in *sch9Δ*, *ras2Δ*, and *tor1Δ *using a cut-off based method [29]. This method applied the Fisher's exact test to examine the enrichment of each motif in the up- and down-regulated gene sets from *sch9Δ/wt*, *ras2Δ/wt*, and *tor1Δ/wt*. Although the selection of cutoff is generally not trivial, in this case the cut-off based method and the BASE method achieve similar results. For example, both Msn2/4 and Gis1 binding motifs are found to be significantly activated or enriched in the up-regulated gene sets in all three long-lived mutants with respect to the wild-type. In addition, we tried the linear regression based method, MOTIF REGRESSOR, which did not identify Msn2/4 and Gis1 as activity changed TFs in these long-lived mutants. In fact, no significant linear relationship between gene expression changes in *sch9Δ/wt*, *ras2Δ/wt*, and *tor1Δ/wt *and the motif matching scores of Msn2/4 or Gis1 is revealed from scatter plots and their correlation coefficients.

### TF activity changes in lung carcinomas

In the third case, we apply our method to study the transcriptional regulation in tumors. We seek to identify TFs that have significantly different activities in small cell lung carcinomas (SMC), squamous cell lung carcinomas (SQ) and pulmonary carcinoids (COID) with respect to normal lung tissues. We use the microarray data set provided by Bhattacharjee *et al. *[30], which includes gene expression profiles in specimens from SMC, SQ, COID and normal tissues. We calculate the t-statistic for each gene, which summarizes the difference of gene expression in SMC, SQ or COID with respect to normal lung tissues. In the mean time, we extract all available PWMs from TRANSFAC [31] and use the MATCH program [32] to calculate their matching-scores in the promoter regions of all human genes. To apply our method, the t-scores are taken as the gene expression change data and the matching-scores are taken as the binding affinity data. We identify all PWMs that are significantly associated with the expression differentiation profiles of lung tumors with respect to normal lung tissues. These PWMs and their associated TFs may reflect the differences in transcriptional regulation between lung tumors and normal lung tissues and thus provide us with biological insights about carcinogenesis.

Table 2 shows 27 PWMs that are significant in SMC, SQ and COID (q-value < 0.1) according to our method. Among these PWMs, 10 are binding motifs of E2F family members or E2F related DNA binding proteins according to TRANSFAC. E2F is a heterodimeric complex that is composed of an E2F-family member (E2F-1, E2F-2, E2F-3, E2F-4) and DP-1. It plays a major role during the G1/S transition in the mammalian cell cycle via regulating the transcription of genes that encode cyclins, CDKs, checkpoints regulators, DNA repair and replication proteins [33,34]. The involvement of E2F family members in cancer has been shown in previous studies [35,36]. Our results show significant positive AC scores of these E2F family members in lung carcinoma (SMC and SQ) and pulmonary carcinoids (COID), and indicate the high rate of proliferation of cells. Moreover, AC scores of these PWMs in carcinomas tend to be higher than those in carcinoids (COID), suggesting more active proliferation of cells in carcinomas. Our results also indicate that the activity of P53 is repressed in SMC, SQ and COID, especially in SQ. P53 is known to be one of the most important tumor suppressor gene that protects humans from cancer. More than half of human cancers harbor p53 mutations and have no functional p53 protein [37-39]. In addition, activities of two other transcription factors, IRF1 and NFKB, are revealed to be repressed in SMC, SQ and especially in COID according to our method. NFKB is a primary transcription factor found in all cell types and is involved in cellular responses to stimuli such as stress, cytokines, free radicals, ultraviolet irradiation, and bacterial or viral antigens [40,41]. IRF1 is important in the regulation of interferons in response to infection by virus and in the regulation of interferon-inducible genes [42-44]. The involvement of NFKB and IRF1 in oncogenesis has been reported in previous studies [45,46]. Further investigation of these identified TFs may provide new insight into the transcriptional regulations during carcinogenesis.

**Table 2:** PWMs significantly associated with gene expression changes in SMC, SQ, and COID in comparison with normal lung tissues.

	SMC/Normal			SQ/Normal			COID/Normal		
Motif Name	AC	p-value	q-value	AC	p-value	q-value	AC	p-value	q-value
V$CETS1P54_01	9.7	0	0	17.8	0	0	11.1	0	0
V$CETS1P54_03	8.5	0	0	15.2	0	0	9.8	0	0
V$E2F_02	9.2	0	0	6.7	0.0022	0.015	5.0	0.033	0.076
V$E2F_Q2	11.0	0	0	9.4	0	0	7.3	0.0031	0.018
V$E2F_Q4	13.6	0	0	9.2	0	0	5.5	0.0073	0.031
V$E2F1_Q3	18.1	0	0	21.1	0	0	12.0	0	0
V$E2F1_Q6	14.9	0	0	13.9	0	0	7.5	0.0008	0.0066
V$E2F1_Q6_01	13.5	0	0	12.5	0	0	6.2	0.0095	0.037
V$E2F1DP1_01	11.1	0	0	10.9	0	0	6.6	0	0
V$E2F1DP1RB_01	10.3	0	0	7.5	0.0013	0.010	5.2	0.031	0.074
V$E2F1DP2_01	10.0	0	0	9.4	0	0	7.0	0.0013	0.0096
V$E2F4DP2_01	9.4	0	0	9.7	0	0	6.2	0.0055	0.025
V$ELF1_Q6	-6.7	0.0052	0.041	-7.9	0	0	-9.3	0	0
V$ELK1_02	6.7	0.0063	0.047	16.1	0	0	9.6	0	0
V$ETF_Q6	6.6	0.013	0.070	5.2	0.017	0.066	7.6	0	0
V$HNF3_Q6	-5.4	0.015	0.079	-5.9	0.031	0.096	-7.8	0	0
V$HNF3_Q6_01	-5.9	0.0094	0.060	-6.4	0.014	0.059	-8.5	0.0001	0.0010
V$IRF1_Q6	-6.4	0.019	0.089	-5.7	0.019	0.070	-9.0	0	0
V$NFKAPPAB65_01	-6.1	0.0067	0.048	-6.0	0.020	0.072	-8.2	0	0
V$NRF1_Q6	7.7	0	0	8.4	0	0	7.4	0.0012	0.0092
V$P53_02	-5.4	0.022	0.099	-6.3	0	0	-4.9	0.041	0.089
V$PAX3_B	6.0	0.018	0.086	7.6	0	0	5.9	0.0094	0.037
V$PEA3_Q6	-7.3	0.0001	0.0010	-6.2	0.0008	0.0077	-13.0	0	0
V$SREBP1_Q6	-7.4	0	0	-12.0	0	0	-8.3	0.0022	0.014
V$STAF_02	8.4	0	0	8.1	0.0013	0.010	8.3	0	0
V$ZF5_01	10.5	0	0	7.3	0.0050	0.028	7.4	0	0
V$ZF5_B	8.4	0	0	6.3	0.0040	0.023	5.8	0.019	0.058

Only the PWMs that are significant (q-value < 0.1) in all the three tumor subtypes are shown.

## Conclusion

We have developed a novel method to infer activity changes of transcription factor by integrating the gene expression data with binding affinity data such as ChIP-chip or motif data. Unlike previous approaches, this method does not assume linear relationship between TF-gene binding affinities and gene expression changes. Since this method does not need pre-defined target gene sets, it requires no threshold selection for binding affinity scores or gene expression changes. This method is applied to three different data sets in which the gene expression data are integrated with ChIP-chip data, motif discovery data and motif scanning data, respectively. The implications obtained from each data set are biologically meaningful and consistent with previous studies. Moreover, the method is robust to noise in expression data and easy to be implemented. Potentially, this method may be applied to many microarray data sets to shed light on the mechanisms of transcriptional regulation.

## Methods

### Significance assessing of activity change

The goal of our method is to infer activity change of a given transcription factor by integrating gene expression data with binding affinity data. Let *e *= (*e*_1_, *e*_2_,⋯, *e*_*N*_) be the expression differentiation vector for the *N *genes on the microarray, where *e*_*i *_describes the gene expression fold change (log ratio) of gene *i *between two conditions. Based on the binding affinity data from ChIP-chip experiments or motif discovery analysis, we extract another vector called binding vector *m *= (*m*_1_, *m*_2_,⋯, *m*_*N*_), where *m*_*i *_measures the binding affinity of the given transcription factor to the upstream region of gene *i*. Here we assume that the expression differentiation vector *e *is already normalized so that it more or less centers around 0 and the values in the binding vector *m *are non-negative. A robust version of binding vector is the replacement of binding affinities by their ranks. However, our computational experience indicates some loss of power from the rank binding vector.

To infer the activity change of the given transcription factor, we first sort the expression differentiation vector into *e*' = (*e*_(1)_, *e*_(2)_,⋯, *e*_(*N*)_), where *e*_(*i*) _≥ *e*_(*i*+1) _for any 1 ≤ *i *≤ *N *- 1. Suppose the corresponding indices of the ranked genes is (*i*_1_, *i*_2_,⋯, *i*_*N*_), that is, gene *i*_*k *_has the *k*-th largest gene expression change.

m′=(mi1,mi2,⋯,miN)
 MathType@MTEF@5@5@+=feaafiart1ev1aaatCvAUfKttLearuWrP9MDH5MBPbIqV92AaeXatLxBI9gBaebbnrfifHhDYfgasaacPC6xNi=xH8viVGI8Gi=hEeeu0xXdbba9frFj0xb9qqpG0dXdb9aspeI8k8fiI+fsY=rqGqVepae9pg0db9vqaiVgFr0xfr=xfr=xc9adbaqaaeGacaGaaiaabeqaaeqabiWaaaGcbaGafmyBa0MbauaacqGH9aqpcqGGOaakcqWGTbqBdaWgaaWcbaGaemyAaK2aaSbaaWqaaiabigdaXaqabaaaleqaaOGaeiilaWIaemyBa02aaSbaaSqaaiabdMgaPnaaBaaameaacqaIYaGmaeqaaaWcbeaakiabcYcaSiabl+UimjabcYcaSiabd2gaTnaaBaaaleaacqWGPbqAdaWgaaadbaGaemOta4eabeaaaSqabaGccqGGPaqkaaa@4113@ and therefore both mij
 MathType@MTEF@5@5@+=feaafiart1ev1aaatCvAUfKttLearuWrP9MDH5MBPbIqV92AaeXatLxBI9gBaebbnrfifHhDYfgasaacPC6xNi=xH8viVGI8Gi=hEeeu0xXdbba9frFj0xb9qqpG0dXdb9aspeI8k8fiI+fsY=rqGqVepae9pg0db9vqaiVgFr0xfr=xfr=xc9adbaqaaeGacaGaaiaabeqaaeqabiWaaaGcbaGaemyBa02aaSbaaSqaaiabdMgaPnaaBaaameaacqWGQbGAaeqaaaWcbeaaaaa@3052@ and *e*_(*j*) _correspond to gene *i*_*j*_. Next, we define a non-decreasing function *f *(*i*) based on the two vectors *e' *and *m' *as follows:

f(i)=∑j=1i|e(j)mij|∑j=1N|e(j)mij|,1≤i≤N.
 MathType@MTEF@5@5@+=feaafiart1ev1aaatCvAUfKttLearuWrP9MDH5MBPbIqV92AaeXatLxBI9gBaebbnrfifHhDYfgasaacPC6xNi=xI8qiVKYPFjYdHaVhbbf9v8qqaqFr0xc9vqFj0dXdbba91qpepeI8k8fiI+fsY=rqGqVepae9pg0db9vqaiVgFr0xfr=xfr=xc9adbaqaaeGacaGaaiaabeqaaeqabiWaaaGcbaGaemOzayMaeiikaGIaemyAaKMaeiykaKIaeyypa0tcfa4aaSaaaeaadaaeWaqaamaaemaabaGaemyzau2aaSbaaeaacqGGOaakcqWGQbGAcqGGPaqkaeqaaiabd2gaTnaaBaaabaGaemyAaK2aaSbaaeaacqWGQbGAaeqaaaqabaaacaGLhWUaayjcSdaabaGaemOAaOMaeyypa0JaeGymaedabaGaemyAaKgacqGHris5aaqaamaaqadabaWaaqWaaeaacqWGLbqzdaWgaaqaaiabcIcaOiabdQgaQjabcMcaPaqabaGaemyBa02aaSbaaeaacqWGPbqAdaWgaaqaaiabdQgaQbqabaaabeaaaiaawEa7caGLiWoaaeaacqWGQbGAcqGH9aqpcqaIXaqmaeaacqWGobGtaiabggHiLdaaaiabcYcaSiabigdaXiabgsMiJkabdMgaPjabgsMiJkabd6eaojabc6caUaaa@5FC3@

As a reference, we define another non-decreasing function *g*(*i*) based only on *e' *itself:

g(i)=∑j=1i|e(j)|∑j=1N|e(j)|.
 MathType@MTEF@5@5@+=feaafiart1ev1aaatCvAUfKttLearuWrP9MDH5MBPbIqV92AaeXatLxBI9gBaebbnrfifHhDYfgasaacPC6xNi=xI8qiVKYPFjYdHaVhbbf9v8qqaqFr0xc9vqFj0dXdbba91qpepeI8k8fiI+fsY=rqGqVepae9pg0db9vqaiVgFr0xfr=xfr=xc9adbaqaaeGacaGaaiaabeqaaeqabiWaaaGcbaGaem4zaCMaeiikaGIaemyAaKMaeiykaKIaeyypa0tcfa4aaSaaaeaadaaeWaqaamaaemaabaGaemyzau2aaSbaaeaacqGGOaakcqWGQbGAcqGGPaqkaeqaaaGaay5bSlaawIa7aaqaaiabdQgaQjabg2da9iabigdaXaqaaiabdMgaPbGaeyyeIuoaaeaadaaeWaqaamaaemaabaGaemyzau2aaSbaaeaacqGGOaakcqWGQbGAcqGGPaqkaeqaaaGaay5bSlaawIa7aaqaaiabdQgaQjabg2da9iabigdaXaqaaiabd6eaobGaeyyeIuoaaaGaeiOla4caaa@4F51@

From these two non-decreasing functions, a statistic, denoted as the pre-score, is calculated to measure the maximum difference between *f*(*i*) and *g*(*i*) as follows:

*ps *= *f*(*i*_*max*_) - *g*(*i*_*max*_)

where imax=arg⁡max⁡i=1,2,⋯,N|f(i)−g(i)|
 MathType@MTEF@5@5@+=feaafiart1ev1aaatCvAUfKttLearuWrP9MDH5MBPbIqV92AaeXatLxBI9gBaebbnrfifHhDYfgasaacPC6xNi=xH8viVGI8Gi=hEeeu0xXdbba9frFj0xb9qqpG0dXdb9aspeI8k8fiI+fsY=rqGqVepae9pg0db9vqaiVgFr0xfr=xfr=xc9adbaqaaeGacaGaaiaabeqaaeqabiWaaaGcbaGaemyAaK2aaSbaaSqaaiabd2gaTjabdggaHjabdIha4bqabaGccqGH9aqpdaWfqaqaaiGbcggaHjabckhaYjabcEgaNjGbc2gaTjabcggaHjabcIha4bWcbaGaemyAaKMaeyypa0JaeGymaeJaeiilaWIaeGOmaiJaeiilaWIaeS47IWKaeiilaWIaemOta4eabeaakmaaemaabaGaemOzayMaeiikaGIaemyAaKMaeiykaKIaeyOeI0Iaem4zaCMaeiikaGIaemyAaKMaeiykaKcacaGLhWUaayjcSdaaaa@51D3@.

The statistic has several features. First, if *e *and *m *are not associated with each other, *f*(·) will be centered around *g*(·), leading to a small value of *ps*. Second, we take the maximum difference between the two functions as our statistic, which overcomes the problem of thresholding on the TF-gene binding affinities. Third, the strengthes of both gene expression changes and binding affinities are considered in our statistic, which makes our statistic more powerful to detect TF activity changes. Fourth, in a special case where binding affinities are treated as 1 if they are equal to or larger than a specified threshold and 0 otherwise, the statistic is similar to the enrichment score used in GSEA [47]. Finally, our definition of binding association with sorted expression is scale-invariant in the sense that the scores remain unchanged if we apply different scales to binding scores of different TFs. This is a desirable property, for binding of TF with DNA is TF-specific.

To assess the significance of *ps*, we permute the reordered binding vector *M *times and obtain *M *permuted binding vectors *m*^(1)^, *m*^(2)^,⋯, *m*^(*M*)^. For each permutation, the *ps *is recalculated by replacing *m' *in equation (1) with the permuted binding vector. This permutation procedure results in *M *permuted *ps *statistics, denoted as *ps*^*perm *^= (*ps*^1^, *ps*^2^,⋯, *ps*^*M*^). Based on these permutated pre-scores, the one-sided p-value for activity change of the given TF in the gene expression experiment is defined as

p={#{i:psi≥ps}M,ps≥MEAN(psperm)#{i:psi≤ps}M,ps≤MEAN(psperm),
 MathType@MTEF@5@5@+=feaafiart1ev1aaatCvAUfKttLearuWrP9MDH5MBPbIqV92AaeXatLxBI9gBaebbnrfifHhDYfgasaacPC6xNi=xI8qiVKYPFjYdHaVhbbf9v8qqaqFr0xc9vqFj0dXdbba91qpepeI8k8fiI+fsY=rqGqVepae9pg0db9vqaiVgFr0xfr=xfr=xc9adbaqaaeGacaGaaiaabeqaaeqabiWaaaGcbaGaemiCaaNaeyypa0ZaaiqabeaafaqaaeGacaaabaqcfa4aaSaaaeaacqGGJaWicqGG7bWEcqWGPbqAcqGG6aGocqWGWbaCcqWGZbWCdaahaaqabeaacqWGPbqAaaGaeyyzImRaemiCaaNaem4CamNaeiyFa0habaGaemyta0eaaiabcYcaSaGcbaGaemiCaaNaem4CamNaeyyzImRaemyta0KaemyrauKaemyqaeKaemOta4KaeiikaGIaemiCaaNaem4Cam3aaWbaaSqabeaacqWGWbaCcqWGLbqzcqWGYbGCcqWGTbqBaaGccqGGPaqkaeaajuaGdaWcaaqaaiabcocaJiabcUha7jabdMgaPjabcQda6iabdchaWjabdohaZnaaCaaabeqaaiabdMgaPbaacqGHKjYOcqWGWbaCcqWGZbWCcqGG9bqFaeaacqWGnbqtaaGaeiilaWcakeaacqWGWbaCcqWGZbWCcqGHKjYOcqWGnbqtcqWGfbqrcqWGbbqqcqWGobGtcqGGOaakcqWGWbaCcqWGZbWCdaahaaWcbeqaaiabdchaWjabdwgaLjabdkhaYjabd2gaTbaakiabcMcaPiabcYcaSaaaaiaawUhaaaaa@7AA0@

where *MEAN*(*ps*^*perm*^) is the mean of *ps*^*perm*^. To correct for the multiple testing errors, we calculate the q-values using the "qvalue" package in "Bioconductor" of R [48].

### Activity change (AC) score calculation

From the assessed significance above, we can decide whether the given transcription factor has a significant activity change in the gene expression experiment. However, a more interesting problem is how the transcription factor affects the gene expressions of its target genes. That is, if the transcription factor is significant, we also want to know whether it down-regulates or up-regulates its target genes. Furthermore, limited by the number of permutations, the permutation test does not give enough accuracy for the significance estimation. Many transcription factors may have the same p-value, although they do not have the same magnitudes in activity change. To address these issues, we define an activity change (AC) score which is negative when its target genes are down-regulated and positive when they are up-regulated. Its absolute value reflects the magnitude of activity change. The AC score is defined as

AC=ps−MEAN(psperm)SD(|psperm|),
 MathType@MTEF@5@5@+=feaafiart1ev1aaatCvAUfKttLearuWrP9MDH5MBPbIqV92AaeXatLxBI9gBaebbnrfifHhDYfgasaacPC6xNi=xI8qiVKYPFjYdHaVhbbf9v8qqaqFr0xc9vqFj0dXdbba91qpepeI8k8fiI+fsY=rqGqVepae9pg0db9vqaiVgFr0xfr=xfr=xc9adbaqaaeGacaGaaiaabeqaaeqabiWaaaGcbaGaemyqaeKaem4qamKaeyypa0tcfa4aaSaaaeaacqWGWbaCcqWGZbWCcqGHsislcqWGnbqtcqWGfbqrcqWGbbqqcqWGobGtcqGGOaakcqWGWbaCcqWGZbWCdaahaaqabeaacqWGWbaCcqWGLbqzcqWGYbGCcqWGTbqBaaGaeiykaKcabaGaem4uamLaemiraqKaeiikaGIaeiiFaWNaemiCaaNaem4Cam3aaWbaaeqabaGaemiCaaNaemyzauMaemOCaiNaemyBa0gaaiabcYha8jabcMcaPaaacqGGSaalaaa@529A@

where *MEAN*(*ps*^*perm*^) is the mean of *ps*^*perm*^and *SD*(|*ps*^*perm*^|) is the standard deviation for the absolute values of *ps*^*perm*^. Basically, the above defined AC score is used to standardize the pre-score by a shift-scale transformation. The location parameter is taken to be the mean of the pre-scores from the permutations. The selection of scale parameter is subtle, since *ps*^*perm *^has a bimodal distribution. It can be shown that the positive pre-scores and the negative pre-scores from the permutations have the same distribution, if the expression change profile *e *is symmetric against zero, which is approximately satisfied for most microarray data. Therefore, we use the standard deviation of the |*ps*^*perm*^| rather than *ps*^*perm *^to represent the variance of the permutated pre-scores. If the given TF is an activator, then a positive AC score and a negative AC score indicate activity enhancement and reduction, respectively. Conversely, if the TF is a repressor, then the inferences of activity change are opposite.

### Integration of TFPE with ChIP-chip data

We collect 76 microarray gene expression change profiles from four groups of transcription factor perturbation experiments (TFPE) in yeast, each measuring gene expression changes in the yeast strain where a single TF is deleted or over-expressed. The 76 gene expression change profiles include 27 TF deletion profiles and 49 TF over-expression profiles. In these 27 deletion profiles, 22 are from Hughes *et al. *[9] and 5 are from Mnamneh *et al. *[10]. In the 49 over-expression of of transcription factors, 46 are from Chua *et al. *[12] and 3 are from *Gasch et al. *[11] (see Additional file 1 and file 2). When we apply BASE to the combined data set, each of the 76 gene expression change profiles is used separately as the expression differentiation vector *e*. The binding affinity data is obtained from the ChIP-chip data reported in Harbison *et al. *[3], which includes 350 ChIP-chip profiles for 203 TFs (some TFs are measured under multiple conditions). Each ChIP-chip profile measures the binding affinities of a TF to the promoter regions of all yeast genes under a specific experimental condition. Each of these profiles is taken as the binding vector *m*. For every combination of the 76 gene expression change profiles and the 350 TF-gene binding affinity profiles, we apply our method to calculate the AC score as well as its significance.

### Integration of Gene Expression with Motif Discovery Data

We measure the expression levels for 5667 yeast genes using Affymetrix Yeast2.0 microarrays in the wild-type and three long-lived yeast mutants: *sch9Δ*, *ras2Δ *and *tor1Δ*. The binding affinity data is calculated based on the motif discovery data published by Beer *et al. *[24]. They identified 666 enriched motifs in the promoter regions (DNA sequences from the translation initiation site to 800 bp upstream) of all yeast genes using AlignACE [23]. The occurrences of each motif in the upstream region of each gene (800 bp) were then determined by searching the motif against these sequences. The motif discovery data contain the number of occurrences and their matching-scores of each motif in the upstream region of each gene (the cut-off for matching-score is set to 0.5). Suppose that for each motif there exists a DNA binding protein (e.g. a TF) associated with it, these motif matching-scores reflect the binding affinities of the protein to the promoters of genes. In this paper, we select 537 from these 666 motifs after calculating their pairwise similarities and removing the redundant ones. Matching-scores of all occurrences for the same motif in the upstream region of a gene are aggregated. When no occurrence is found in the upstream of a gene, the score is set to 0. The above described calculations result in an aggregated matching-score for each pair of motif and gene, which reflects the binding affinity of the corresponding TF to this gene via that motif. To apply our method, the vector containing the aggregated matching-scores of a motif in the upstream regions of all genes is taken as the binding vector *m*, and each of the expression change profiles for *sch9Δ/wt*, *ras2Δ/wt *and *tor1Δ/wt *is treated as the expression differentiation vector *e*. For each of the 537 motifs, the BASE method is used to integrate its binding vector with each of the three expression change profiles. The AC scores and their significance are calculated for all 537 motifs in *sch9Δ/wt*, *ras2Δ/wt *and *tor1Δ/wt*.

### Integration of Gene Expression with PWM Scanning Data

In the study reported in Bhattacharjee *et al. *[30], gene expression levels in 6 small-cell lung carcinomas (SMC), 21 squamous cell lung carcinomas (SQ), 20 pulmonary carcinoids (COID) and 17 normal lung specimens were measured. We calculate the t-statistic for all the genes using the 6 SMCs, 21 SQs or 20 COIDs versus the 17 normal samples, resulting in three t-score profiles for SMC/normal, SQ/normal, and COID/normal, respectively. Each of these profiles is taken as the expression differentiation vector *e*. The binding affinity data is calculated based on the 546 positional weight matrices (PWMs) in vertebrates extracted from TRANSFAC9.4 [32]. For each of these 546 PWMs, we used the program MATCH to scan the upstream regions of all human genes from the transcription start site up to 1000 bp [31]. To minimize the false positive rate, the pre-calculated cut-off values for these PWMs (provided by the MATCH program) are used. The matching-scores for all significant hits of the same PWM in each upstream region are aggregated. When no hit is found in the upstream region of a gene, the score is set to 0. The vector of the aggregated matching-scores for each PWMs is taken as the binding vector *m*. The above data processing results in 3 expression change vectors (SMC, SQ, and COIDs) and 546 binding vectors. For each combination of expression change profiles and matching-score vectors, we applied our method to calculate the AC score as well as its significance.

## Authors' contributions

CC and XY designed the method, wrote the code, carried out the analysis, and drafted the manuscript. FS and LL participated in the design and the coordination of the study. All authors read and approved the final manuscript.

## Supplementary Material

Additional file 1 AC scores and their significance for perturbed TFs in deletion TFPEs. Click here for file

Additional file 2 AC scores and their significance for perturbed TFs in over-expression TFPEs. Click here for file

Additional file 3 Significance of TF activity changes in deletion TFPEs inferred by using Tsai et al.'s method [8]. Click here for file

Additional file 4 Significance of TF activity changes in over-expression TFPEs inferred by using Tsai et al.'s method [8]. Click here for file

Additional file 5 AC scores and their significance for the 537 motifs in the long-lived mutants. These motifs are identified from promoter regions of all yeast transcripts by Beer et al. [24]. Click here for file
